# Initial Liver Copper Status in Finishing Beef Steers Fed Three Dietary Concentrations of Copper Affects Beta Agonist Performance, Carcass Characteristics, Lipolysis Response, and Muscle Inflammation Markers

**DOI:** 10.3390/ani11092753

**Published:** 2021-09-21

**Authors:** Elizabeth Messersmith, Mark Branine, Olivia Genther-Schroeder, Jodi McGill, Stephanie Hansen

**Affiliations:** 1 Department of Animal Science, Iowa State University, Ames, IA 50011, USA; emm2@iastate.edu (E.M.); genthero@alumni.iastate.edu (O.G.-S.); 2 Zinpro Corporation, Eden Prairie, MN 55344, USA; mbranine@zinpro.com; 3 Department of Veterinary Microbiology and Preventative Medicine, Iowa State University, Ames, IA 50011, USA; jlmcgill@iastate.edu

**Keywords:** cattle, feedlot, lipid metabolism, ractopamine hydrochloride, trace mineral

## Abstract

**Simple Summary:**

Beta agonists are commonly used in the United States beef industry, offering improved performance in the days leading up to harvest by influencing energy metabolism. Copper has been shown to regulate the biological pathway leading to increased lipid mobilization. However, this connection has not been evaluated in cattle. Therefore, the objective of this study was to determine how Cu influences beta agonist-induced performance, energy metabolism and inflammation in feedlot cattle. Supplementation of Cu resulted in increased liver Cu concentrations, while cattle performance, lipolysis, and some markers of inflammation responded to Cu supplementation differently, depending on whether or not cattle were fed a beta agonist. Therefore, strategic supplementation of Cu may help optimize growth of cattle receiving a beta agonist.

**Abstract:**

Ninety-three Angus-crossbred steers (470 ± 35 kg) were assigned to a 3 × 2 factorial to determine the effects of Cu status and beta agonist (BA) on performance, carcass characteristics, lipolytic rate, and muscle inflammation. Factors included Cu supplementation (mg Cu/kg dry matter (DM)) at: 0 (LO), 10 (MED), or 20 (HI) from Cu amino acid complex (Availa Cu; Zinpro) with no BA (NoRAC) or 300 mg·steer^−1^·day^−1^ of ractopamine hydrochloride (RAC; Optaflexx; Elanco) for final 28 days of 88-day trial. Linear and quadratic effects of Cu status within BA treatment were tested. Pre-BA gain was not affected by Cu supplementation (*p* ≥ 0.57), although day 53 liver Cu quadratically increased (*p* = 0.01). Average daily gain and muscle IL-8 gene expression quadratically increased (*p* ≤ 0.01), with MED having greatest gain and gene expression. Ribeye area tended to quadratically increase with Cu supplementation within RAC (*p* = 0.08). In vitro basal lipolytic rate tended to quadratically increase with Cu supplementation within RAC (*p* = 0.11), while stimulated lipolytic rate tended to linearly increase within NoRAC (*p* = 0.10). These data suggest lipolysis and the BA response of steers are influenced by dietary and liver Cu concentrations.

## 1. Introduction

Cattle have historically been considered a Cu-tolerant species capable of homeostatic control through hepatic Cu storage and biliary Cu excretion [1]. However, Cu deficiency [2,3] and toxicity [1,4] concerns are still prevalent in both the beef and dairy industry, indicating dietary Cu concentrations should be more carefully evaluated. Currently, Cu supplementation strategies for beef cattle rely on the National Academies of Sciences, Engineering, and Medicine (NASEM) [5] recommendation of 10 mg Cu/kg dry matter (DM) developed to prevent Cu deficiency but surveyed consulting nutritionist recommendations vary broadly, from 10 to 40 mg/kg DM of supplemental Cu for finishing cattle [6]. Trace mineral recommendations are interpreted as dietary concentrations required, which are not often analyzed. Additionally, the current reference range for adequate liver Cu concentration is wide (125 to 600 mg Cu/kg DM) [7], thereby limiting the interpretation of reference ranges to determine optimal Cu supplementation.

Biological roles of Cu are abundant, including collagen cross-linking by Cu-dependent lysyl oxidase [8] and serving as a cofactor in the antioxidant Cu/Zn superoxide dismutase [9]. Beta agonists work through G protein-coupled receptors that induce cyclic adenosine monophosphate (cAMP) production and the release of glycerol and non-esterified fatty acids (NEFA) [10]. Copper has been shown to positively influence lipolysis in mouse and cell culture models by hindering phosphodiesterase (PDE) inhibition of cAMP [11] and therefore the subsequent production of glycerol and NEFA. Utilizing these lipid stores is a key component of the beta agonist (BA) mode of action [10]. Therefore, Cu status may influence lipolysis of BA-fed cattle, leading to an influx of energy substrates available for lean tissue accretion during the BA feeding period. The objective of this study was to determine if performance, carcass characteristics, and lipolytic rate of beef steers were differentially affected by three distinct liver Cu concentrations achieved through dietary Cu supplementation when a BA was fed. We hypothesized that increasing Cu supplementation would improve BA performance and lipolytic rate of BA-fed steers, while no differences in performance were expected due to increasing rate of Cu supplementation of late-stage finishing steers not receiving a BA.

## 2. Materials and Methods

### 2.1. Care and Use of Animals

All procedures and protocols in this study were approved by the Iowa State University Institutional Animal Care and Use Committee (IACUC; log number 5-17-8522-B).

### 2.2. Experimental Design

Ninety-three single source Angus-crossbred steers (initial body weight (BW) = 470 ± 35 kg) were available for use in a 3 × 2 randomized incomplete block design experiment. These steers were utilized in a previous trial in which 3 distinct liver Cu statuses were induced through the dietary inclusion of Cu antagonists S and Mo [12]. At the conclusion of the prior 85-day experiment, steers were sorted by liver Cu concentration (average liver Cu concentrations of 6, 14, and 53 mg Cu/kg DM for LO, MED, and HI, respectively) into 3 dietary treatments to maintain liver Cu distinctions throughout the current trial. Sorting by liver Cu concentrations resulted in the majority of steers remaining on the previous trial’s Cu groupings with only 3 to 6 steers moving to an adjacent Cu treatment for LO, MED, and HI. Liver Cu concentrations analyzed from the end of the prior experiment were utilized as initial liver Cu concentrations for the current trial. During the 23 day between the end of the previous trial and the start of the current trial, cattle were maintained on their previous Cu treatment, a small timeframe not likely to have influenced liver Cu concentrations greatly. Dietary treatments were initiated on day 0 and included LO, MED, and HI receiving 0, 10, or 20 mg of supplemental Cu/kg DM from Cu amino acid complex (Availa Cu; Zinpro Corporation, Eden Prairie, MN, USA), respectively. These treatments represent no supplemental Cu (LO), NASEM [5] recommendations (MED), and industry-consultant-reported [6] supplementation (HI). Within Cu treatment, steers were stratified by initial BW and randomly assigned to pens of 5 or 6 steers (LO: *n* = 4 pens MED: *n* = 6 pens, HI: *n* = 6 pens). Each pen was equipped with an automatic waterer and a single GrowSafe bunk (GrowSafe Systems Ltd., Airdrie, AB, Canada); individual steer’s radio frequency tags were linked to GrowSafe software to record individual steer feed disappearance. As-fed feed disappearance was corrected for DM to calculate individual steer dry matter intake (DMI). Therefore, for the Pre-BA period, *n* = 24, 33, 34 steers for LO, MED, and HI, respectively.

Consecutive day BW were taken at the beginning of the Pre-BA period (day −1 and 0), the beginning of the BA period (day 60 and 61), and at the end of the experiment (day 87 and 88). Cattle were implanted on day 0 with a Component TE-IS (Elanco Animal Health, Greenfield, IN) followed by a Component TE-S (Elanco Animal Health) on day 40. Albeit close in timing, the terminal Component TE-S implant was administered on day 40 to ensure steers would benefit from an implant throughout the entirety of the trial and prevent re-implanting too close to harvest. On day 61, half of the pens from each dietary Cu treatment were randomly assigned to receive a BA (ractopamine hydrochloride; Optaflexx, Elanco Animal Health) at 0 (NoRAC) or 300 (RAC) mg·steer^−1^·day^−1^ for the final 28 days of the trial. Supplemented BA inclusion was back-calculated using individual steer intakes; across treatments, BA inclusion averaged 310 mg·steer^−1^·day^−1^ (Standard deviation = 35.0). At the start of the BA period, *n* = 12, 12, 16, 17, 18, and 16 for LO-RAC, LO-NoRAC, MED-RAC, MED-NoRAC, HI-RAC, and HI-NoRAC, respectively.

Cattle were fed a common dry rolled corn-based diet supplemented with NASEM [5] recommendations for all minerals, except Cu, from inorganic sources (Table 1). Dietary Cu concentrations analyzed as 6.1, 15.2, and 27.3 mg Cu/kg DM during the Pre-BA period and 6.0, 17.3, and 22.0 mg Cu/kg DM during the BA period for LO, MED, and HI treatments, respectively. Copper and BA treatments were administered through the diet using a dried distiller’s grain basal supplement incorporated into the total mixed ration (TMR). Diets were fed from lowest Cu inclusion to highest within each BA treatment with NoRAC fed before RAC. The mixer was flushed after feeding RAC diets. During the Pre-BA period, an error in DM was recognized for modified distiller’s grains with solubles, and it was found to be most appropriate to correct upon the start of the BA period. Thus, steers received the Pre-BA diet from day 0–60 and the BA diet from day 61–88. The diet was delivered at approximately 0800 h daily, and bunks were managed to allow for ad libitum feed intake.

Cattle were shipped to a commercial abattoir (Iowa Premium Beef, Tama, IA, USA) on day 88 and were harvested on day 89 using the industry-accepted method of captive bolt. Carcasses were chilled for 48 h before ribbing. Carcass data were collected, including hot carcass weight (HCW), ribeye area (REA), rib fat thickness (RF), kidney, pelvic, heart fat (KPH), and marbling score, and yield grade (YG) was calculated. Additionally, dressing percentage (DP) was calculated by dividing the shrunk live final BW by the HCW and multiplying by 100. A 4% pencil shrink was applied to all BW before average daily gain (ADG) and gain-to-feed ratio (G:F) were calculated.

### 2.3. Sample Collection and Analysis

Total mixed rations were sampled weekly and dried in a forced air oven at 70 °C for 48 h to determine DM. Utilizing the weekly DM from TMR samples, individual steer DMI was ascertained from as-fed feed disappearance recorded by GrowSafe bunks. Feed efficiency (G:F) was calculated for both the pre-BA and BA periods using total weight gained in that period divided by the total amount of feed consumed on a DM basis during that period. Dried diet samples were ground through a 2-mm screen (Retsch Zm100 grinder; Glen Mills Inc., Clifton, NJ, USA), and samples were composited monthly in accordance with the pre-BA and BA periods.

Liver biopsies were conducted on 3 randomly sampled steers from each pen (*n* = 6 steers per LO and 9 steers per MED and HI treatment) on day −23 and 53 in accordance with the method described by Engle and Spears [13], while final liver samples were collected at harvest. The same 3 steers were utilized for liver samples throughout the experiment. Steers were randomly selected, irrespective of liver samplers, for adipose biopsies (*n* = 4 per treatment) collected from the tailhead, and muscle biopsies (*n* = 4 steers per LO and 6 steers per MED and HI treatment) collected from the longissimus thoracis between the 11th and 12th rib were performed on day 66 or 67 (half of the samples collected each day) following adapted procedures from Koltes and Spurlock [14] and Pampusch et al. [15], respectively. Prior to each biopsy procedure, steers were administered either 3 mL of Lidocaine (2%; VetOne, Boise, ID, USA) for liver biopsies or 10 mL for muscle and adipose biopsies as a local anesthetic in accordance with the approved IACUC procedure.

Adipose samples were utilized for in vitro analysis of lipolytic rate, with procedures adapted from Pothoven et al. [16]. Briefly, approximately 200 mg of adipose tissue was placed in 3 mL of Krebs Ringer Bicarbonate-based buffer for 1 h incubation at 37 °C. In vitro analysis of adipose tissue from each steer was performed in triplicate for both the basal and epinephrine-stimulated buffer. Glycerol concentration was analyzed via gas chromatography-mass spectrometry (GC-MS; Agilent Technologies Model 6890 GC coupled to Model 5975 MS) at the W.M. Keck Metabolomics Research Laboratory (Iowa State University, Ames, IA, USA). In brief, cold methanol was added 1:1 to sample and vortexed then left on ice for 10 min. Next, samples were vortexed before and after sonication on high for 10 min. Samples were centrifuged for 10 min at 10,000× *g* to form a pellet, and the supernatant was transferred to a glass GC vial. The above steps were repeated on the remaining sample pellet, and the second supernatant was pooled with the first and concentrated utilizing nitrogen gas. The concentrated sample was dried in a speed vacuum overnight prior to storage at −80 °C until derivatization for GC-MS analysis. Before sample analysis, acetonitrile solvent was added to each sample with subsequent vortex (5 min) and sonication (10 min). Derivatization occurred with the addition of N, O-Bis(trimethylsilyl)trifluoroacetamide via sialylation at 65 °C for 1 h. Prepped samples were analyzed with helium gas as a carrier through an Agilent-HP5MSI (30 m long, 0.25 mm ID, and 0.25 μm film thickness). The initial oven temperature was 80 °C for 1 min followed by 10 °C/min ramp to 210 °C and 25 °C/min ramp to 320 °C with a final hold for 5 min. Inlet and interface temperatures remained steady at 280 °C. The detection mass range was set from 40–800 m/z. The GC-MS instrument was controlled by Agilent ChemStation software, and glycerol was identified using total ion mass spectrum and comparison to NIST 14 library [17]. An internal standard was used to quantify glycerol concentrations. Samples were evenly distributed by treatment between four GC-MS runs that were completed on the same instrument over the course of one month.

Muscle samples were snap-frozen with liquid nitrogen and stored at −80 °C until quantitative real-time polymerase chain reaction (qPCR) analysis was undertaken for carnitine palmitoyl-CoA transferase-1 (CAT-1) and markers of inflammation, including interleukin-15 (IL-15), interleukin-15α (IL-15α), cyclin-dependent kinase 11B (CD11B), cluster of differentiation 68 (CD68), interleukin-8 (IL-8), chemokine C-X-C motif receptor 1 (CXCR1), and chemokine C-X-C motif receptor 2 (CXCR2). Methods utilized for sample preparation and the reaction cycling conditions were as described by McGill et al. [18], with the reaction taking place in a QuantStudio3 Realtime PCR machine (Applied Biosystems, Life Technologies, Carlsbad, CA, USA). Briefly, RNA was isolated from muscle tissue using Trizol Reagent (Invitrogen, Waltham, MA, USA, Life Technologies, Carlsbad, CA, USA) and cDNA was synthesized using random primers and Superscript III Reverse Transcriptase (Invitrogen, Waltham, MA, USA, Life Technologies, Carlsbad, CA, USA) in accordance with manufacturer’s instructions. Power SYBR Green PCR Master Mix (Applied Biosystems, Carlsbad, CA, USA) was utilized to run qPCR. The amplification conditions were as follows: 2 min at 50 °C, 10 min at 95 °C, 40 cycles of 15 s at 95 °C, and 1 min at 60 °C. The dissociation step included 15 s at 95 °C, 1 min at 60 °C, 15 s at 95 °C, and 15 s at 60 °C. Primers utilized for qPCR are included in Table 2. The housekeeping gene ribosomal protein S9 (RPS-9) was utilized as a reference to determine relative gene expression of each parameter utilizing the 2^−∆∆Ct^ method [19] and calculations of mRNA relative expression were conducted relative to LO-NoRAC.

Jugular blood samples were collected from all animals on day 0, 60, 66/67, 75, and 87. Blood samples were collected in vacutainer tubes with no additives for serum and trace mineral grade K_2_EDTA (Becton Dickenson, Rutherford, NJ, USA). Tubes were centrifuged at 1200× *g* for 10 min for plasma and at 1200× *g* for 20 min for serum samples. Serum and trace mineral plasma were aliquoted and stored at −20 °C prior to analysis. Serum NEFA concentrations were analyzed using a commercial kit (Wako Pure Chemical Industries, Ltd., Chuo-Ku Osaka, Japan) with an intra-assay and inter-assay CV of 4.93% and 13.56%, respectively. Serum urea nitrogen (SUN) concentrations were analyzed utilizing Urea Nitrogen Reagent (Colorimetric Method, Teco Diagnostics, Anaheim, CA, USA) with an intra-assay and inter-assay coefficient of variation of 6.89% and 7.56%, respectively.

Composited TMR and liver samples were acid digested to 20% trace mineral grade nitric acid (Fisher Scientific, Fair Lawn, NJ, USA) with deionized water in accordance with Richter et al. [20] and Pogge and Hansen [21], respectively. Liver and plasma Cu concentrations, as well as TMR Cu and Zn concentrations, were analyzed using inductively coupled plasma optical emission spectrometry (Optima 7000 DV, Perkin Elmer, Waltham, MA, USA) as previously described by Pogge and Hansen [21] and Richter et al. [20]. The S content of TMR was calculated using NASEM [5]-reported nutrient values of diet ingredients.

### 2.4. Statistical Analysis

Performance and intake data from the Pre-BA period (day 0–60) were analyzed using the mixed procedure of SAS 9.4 (SAS Inst. Inc., Cary, NC, USA) with a model including the fixed effect of Cu. Data from two steers (one from MED and HI) were removed from analysis due to poor performance and health concerns unrelated to treatment. Steer was the experimental unit (*n* = 24, 33, or 34 for LO, MED, and HI treatments, respectively) and contrast statements to test for linear or quadratic effects of Cu treatment were utilized. Performance, intake, and carcass characteristics from the BA period (day 61–88) were analyzed as a 3 × 2 factorial using the mixed procedure of SAS 9.4 (SAS Inst. Inc., Cary, NC, USA) using the fixed effects of Cu, BA, and Cu × BA with steer as the experimental unit. Data from the two steers removed during the pre-BA period were also removed during the BA period (one from MED-RAC and one from HI-NoRAC). Thus, *n* = 12 for LO-RAC and LO-NoRAC; 16 for MED-RAC; 17 for MED-NoRAC; 18 for HI-RAC; and 16 for HI-NoRAC for final performance, blood, and carcass data. Contrast statements were formed to test for both linear and quadratic effects of Cu treatment within cattle that did not receive a BA and within cattle that did receive a BA. Proc corr was utilized to test the correlation between individual Cu intake and change in liver Cu concentrations in all liver samplers. Serum NEFA and SUN data were analyzed as repeated measures with day of sampling as the repeated effect and plate as a random effect. Run (on GC-MS) or plate was treated as a random effect for glycerol analysis (*n* = 4 per treatment for glycerol) and muscle CAT1 relative gene expression (*n* = 4 for LO treatments or 6 for all other treatments), respectively. Initial liver Cu from the prior trial (day −119) and day 0 plasma Cu were utilized as covariates in liver (*n* = 6 per LO treatment or 9 per MED and HI treatment) and plasma Cu analysis, respectively. Liver Cu and serum NEFA data were log transformed to fit a normal distribution based on Shapiro–Wilk test. In addition, Cook’s D was used to evaluate data for outliers with Cook’s D values ≥ 0.5 removed from analysis (CAT1: 1 for HI-NoRAC; Plasma Cu: 2 for MED-RAC and HI-RAC (1 of each treatment on day 0 and 66 or 67); in vitro basal lipolytic rate: 1 for MED-NoRAC; NEFA: 1 for MED-RAC (day 75); SUN: 1 for LO-NoRAC, 1 for MED-RAC, and 1 for HI-NoRAC (day 75); CXCR1: 1 MED-RAC; CD68: 1 HI-NoRAC; and IL-15α: 1 for LO-NoRAC). Data reported are least square means with SEM. Both means and SEM were back-transformed and reported where appropriate. Statistical significance was determined at *p* ≤ 0.05, and a statistical tendency was determined at 0.05 < *p* ≤ 0.15.

## 3. Results and Discussion

The body of literature linking trace minerals and growth promoting technologies has been growing over the past decade, signifying the importance of strategic trace mineral supplementation programs in the feedlot. For instance, supplemental Zn has been observed to affect BA-induced performance in cattle [22,23] and pigs [24], while feed conversions in pigs fed BA were improved with Cu supplementation [24]. Relative to Cu, Zn supplementation has been more extensively studied in combination with BA use. However, the unreliability of Zn status markers [1] makes it unclear whether Zn status of animals influences the BA response. Although established reference ranges could be further refined, liver Cu concentrations are an accepted mineral (Cu) status index [7]. Therefore, this study was designed to assess the effect of liver Cu concentrations on BA-induced growth, carcass characteristics, and lipolytic rate.

Although NASEM [5] recommendations for Cu indicate 10 mg/kg DM of dietary Cu is adequate for cattle, the current study supplemented Cu at 0, 10, or 20 mg Cu/kg DM regardless of dietary Cu concentrations (6.1 or 6.0 mg Cu/kg DM). Considering the importance of trace minerals in numerous physiological roles, trace minerals are commonly supplemented at recommendations to ensure adequacy rather than taking fluctuating basal dietary concentrations into account. In agreement with previous literature [25,26,27], the current trial observed no differences due to Cu supplementation on steer BW, ADG, or feed efficiency (Table 3; *p* ≥ 0.32) throughout the Pre-BA period (d 0 to 61).

Pre-BA period DMI tended to linearly increase with increasing Cu supplementation (*p* = 0.15). Interestingly, this tendency for a linear increase in DMI continued throughout the BA period within NoRAC (Table 4; *p* = 0.12) while Cu supplementation did not influence live or carcass-adjusted ADG within NoRAC steers (*p* ≥ 0.39). However, BA period G:F linearly decreased within NoRAC (*p* = 0.04) with higher intakes driving this inefficiency. In contrast, within RAC supplemented steers, DMI during the BA period tended (*p* = 0.10) to quadratically increase due to Cu supplementation, with RAC-MED steers having greater DMI than their counterparts. Additionally, RAC supplementation quadratically improved ADG (*p* = 0.01) with increasing dietary Cu, again driven by RAC-MED steers, resulting in a tendency (*p* = 0.08) for a quadratic increase in G:F due to Cu supplementation of RAC-fed steers.

Furthermore, REA of RAC-fed steers tended (*p* = 0.08) to quadratically increase with RAC-MED steers having the largest REA, while no impact of Cu supplementation within NoRAC steers on REA was noted (Table 5; *p* ≥ 0.38). These data suggest a differential response to Cu supplementation within each BA treatment and imply strategic Cu supplementation may benefit the performance of cattle receiving a BA. However, more work is warranted to determine optimal Cu supplementation strategies considering final live BW, carcass-adjusted final BW, DMI, ADG, and G:F; furthermore, HCW, DP, marbling, RF, KPH, and YG were not affected by Cu supplementation, regardless of BA treatment (*p* ≥ 0.19).

Due to the many biological functions of Cu, assessing Cu status of cattle through liver and plasma is important in determining the physiological condition of the animal as Cu deficiency leads to weakened bones, cardiovascular issues, and ultimately poor growth rates [28]. By design, Pre-BA period dietary Cu intake and initial liver Cu concentrations of steers linearly increased with greater Cu supplementation (Table 3; *p* ≤ 0.01) and day 0 plasma Cu was quadratically increased with Cu treatment (*p* = 0.01). These data corroborate the positive correlation between individual steer Cu intake and the change in liver Cu concentration from the beginning to the end of the trial (*r* = 0.74; *p* < 0.0001). Although steers started this trial with distinct Cu statuses, all 3 treatments were categorized within the deficient (<33 mg Cu/kg DM) or marginal (33 to 125 mg Cu/kg DM) range of Cu status based on liver Cu concentrations as proposed by Kincaid [7]. By the end of the Pre-BA (d 53) period liver Cu concentrations quadratically increased with greater concentrations of supplemental Cu (*p* = 0.01), but no differences were observed in day 60 plasma Cu (*p* ≥ 0.26). Although initially deficient, the rapid increase in liver Cu concentration by day 53 in MED and HI steers may have been enhanced potentially by a lesser physiological need for Cu in late stage finishing cattle in addition to the transition to a high concentrate finishing diet, likely resulting in a drop in ruminal pH, allowing for greater solubility of Cu in the rumen [5].

By design, BA period dietary Cu intake linearly increased with increasing Cu supplementation for both NoRAC and RAC treatments (*p* ≤ 0.01; Table 5), though RAC dietary Cu intake also quadratically increases (*p* = 0.03). This quadratic response is likely a function of the large increase in Cu intakes between treatments. Interestingly, when looking at BA period Cu status day 66 plasma Cu concentrations revealed no linear or quadratic responses (*p* ≥ 0.17) to Cu supplementation within either NoRAC or RAC. However, day 87 plasma Cu concentrations linearly increased with greater Cu supplementation within NoRAC (*p* = 0.03) and tended (*p* = 0.08) to quadratically increase within RAC with maximum plasma Cu concentrations reached with supplementation of MED and HI. Harvest liver Cu concentrations were quadratically increased due to greater Cu supplementation, regardless of BA treatment (*p* = 0.01). These quadratic responses were likely driven by the large incremental differences in liver Cu concentrations between LO, MED, and HI. As such, LO steers had minimal improvements in liver Cu concentrations throughout the study in comparison to MED and HI steers. The inability of LO steers to improve deficient liver Cu concentrations throughout the trial suggest that ~6.0 mg Cu/kg DM analyzed in the common diet was not enough to improve Cu concentration in the liver to the range of adequacy (125 to 600 mg Cu/kg DM) proposed by Kincaid [7]. Furthermore, the inclusion of modified distiller’s grains with solubles resulted in a calculated dietary S concentration of 0.27%, which is likely sufficient to interfere with Cu absorption [29], resulting in the inability of LO steers to improve liver Cu concentrations to the same degree as MED and HI steers. However, the improvement in plasma Cu from deficient (<0.5 mg/L) to adequate (0.7–0.9 mg/L) [7] status for LO steers during the 60-day Pre-BA period suggests these cattle were meeting physiological requirements for Cu regardless of relatively low liver Cu stores. These data indicate deficiency reference ranges for liver Cu concentrations may not be applicable in late-stage finishing steers, as previously thought. Moreover, cattle likely require less dietary Cu than NASEM [5] recommendations (10 mg Cu/kg DM) when dietary Cu antagonists are not in excess as has been suggested by others [25,30].

Moreover, the three distinct Cu statuses observed in the Cu treatments allow for differences in performance and carcass characteristics to be attributed to Cu status based on liver Cu concentrations. Both MED (230 mg Cu/kg DM) and HI (306 mg Cu/kg DM) treatments, regardless of BA treatment, had adequate liver Cu concentrations (125–600 mg Cu/kg DM as suggested by Kincaid [7]) during the BA period. Interestingly, some individual liver Cu concentrations for HI steers were near the top of this wide adequacy range, or even exceeded 600 mg Cu/kg DM. Furthermore, post hoc review of BA period ADG revealed LO steers exhibited roughly no BA response (−2.5%) while MED and HI steers had a 22.8% and 8.3% improvement in ADG due to BA, respectively, though the BA response on average was 9.6%. This clear improvement in performance of RAC-MED steers over RAC-HI during the BA feeding period may suggest that liver Cu concentrations of ~250 mg Cu/kg DM are more favorable than those in excess of 300 mg Cu/kg DM. However, satisfactory growth rates observed across all treatments during the BA period suggest liver Cu adequacy and dietary supplementation of Cu should be further classified to reflect optimal growth, with or without use of growth promoting technologies. Furthermore, the limited number of cattle represented in each treatment indicate a need for additional studies to verify the effects of Cu status on BA-induced performance.

Interestingly, liver Cu concentrations may be linked to efficiency of energy utilization in cattle. Evidence of a relationship between CAT-1, an enzyme involved in the transport of palmitoyl-CoA from the cytosol to the mitochondrial matrix for energy production through β-oxidation [31], and Cu has been detected. Copper deficiency has been observed to decrease hepatic CAT-1 protein abundance in rats [32], while Cu supplementation led to an increase in liver and muscle CAT-1 gene expression in rabbits [33]. The aforementioned literature would suggest liver Cu concentrations may influence the utilization of liberated free fatty acids from BA. However, no linear or quadratic (*p* ≥ 0.75; SEM = 0.290) effects due to Cu supplementation within NoRAC or RAC treatments were observed for CAT-1 relative gene expression in the current study: NoRAC-LO (1.00), NoRAC-MED (1.00), NoRAC-HI (1.04), RAC-LO (0.93), RAC-MED (0.89), and RAC-HI (0.88).

Basic research has revealed Cu is positively associated with lipolytic activity through the reversible regulation of PDE in adipocytes [11]. In brief, PDE acts as a negative effector of cAMP, regulating the BA signaling cascade and the subsequent production of glycerol and NEFA. The regulation of PDE by Cu would suggest a surge in lipolysis with increasing Cu supplementation supported by the decrease in RF observed with increasing Cu supplementation between 10 and 40 mg Cu/kg DM in work by others [26,27,30,34,35]. However, neither Cu or BA impacted RF measures in the present trial.

**Figure 1:**
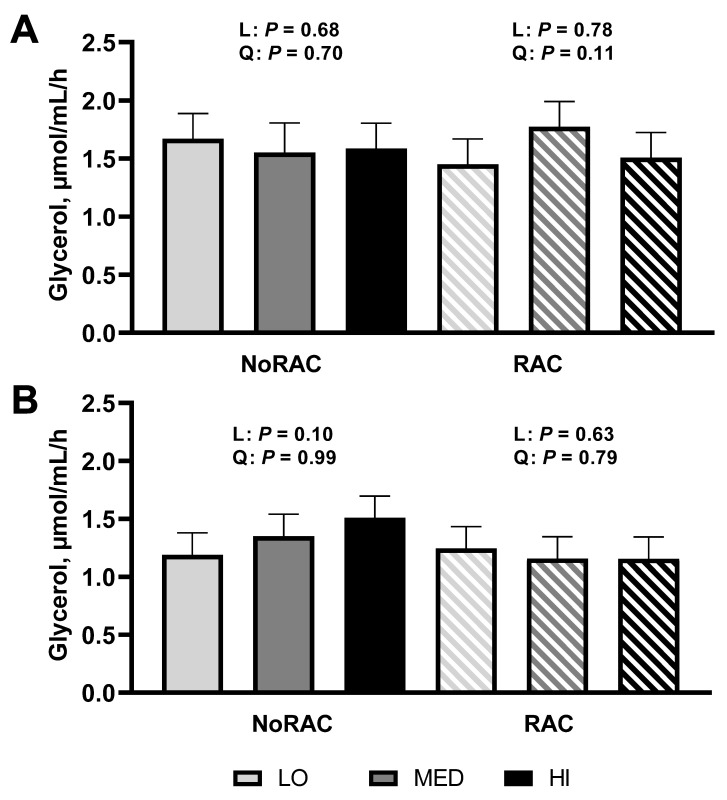
In vitro lipolytic rate of adipose tissue from steers fed 0, 10, or 20 mg Cu/kg DM for LO, MED, and HI, respectively, and 0 (NoRAC) or 300 (RAC) mg·steer^−1^·day^−1^ of ractopamine hydrochloride. Adipose samples were collected on day 66 or 67 (day 6 or 7 of RAC feeding). (**A**) Basal (unstimulated) in vitro lipolytic rate of adipose tissue tended to quadratically increase due to Cu supplementation within RAC steers (*p* = 0.11). No further linear or quadratic effects were observed (*p* ≥ 0.68). (**B**) Epinephrine stimulated in vitro lipolytic rate tended to linearly increase with Cu supplementation within NoRAC (*p* = 0.10). No other linear or quadratic responses were observed (*p* ≥ 0.63) in stimulated lipolysis. Effects of Cu within NoRAC are denoted as L (linear) and Q (quadratic) and effects of Cu within RAC are denoted as L-R (linear) and Q-R (quadratic).

Analysis of glycerol to determine basal (unstimulated) and stimulated lipolytic rate of subcutaneous adipose tissue sampled from steers on day 66 or 67 (day 6 or 7 of BA treatment) revealed a tendency for a quadratic increase due to Cu supplementation within RAC (*p* = 0.11; Figure 1A) with RAC-MED having greatest measures of basal (unstimulated) lipolysis. This agrees with performance data, suggesting these cattle were able to liberate more lipids and subsequently utilize circulating lipids to support growth. However, when adipose was stimulated with epinephrine there was a tendency for glycerol release to linearly increase within NoRAC (*p* = 0.10; Figure 1B) though no other linear or quadratic responses for either basal or stimulated lipolysis were observed (*p* ≥ 0.63), thus suggesting BA-exposed cells may not be able to exhibit further lipolysis through stimulated medium. Balkin and Sonenberg [36] demonstrated pre-exposure of adipocytes to the catecholamine isoproterenol (10^−7^ or 10^−5^ M) or adrenocorticotropic hormone (250 mU/mL) for 2 h resulted in stunted lipolysis upon re-stimulation with either stimulant measured through glycerol release and cAMP in the media, consistent with the improved lipolysis of NoRAC with increasing Cu supplementation and ablated lipolytic response in RAC. These data provide preliminary evidence that the quadratic growth response observed is in part due to the lipolytic rate as affected by dietary Cu supplementation or Cu status of steers (as assessed by liver Cu concentrations). It is unclear why supplementing 10 mg Cu/kg DM improved BA performance more than 20 mg Cu/kg DM. One theory is that supplementing 20 mg Cu/kg DM may have led to greater BA response initially, thus causing faster desensitization of the beta-adrenergic receptor in comparison to lesser concentrations of supplemental Cu, but research is needed to test this hypothesis.

**Figure 2:**
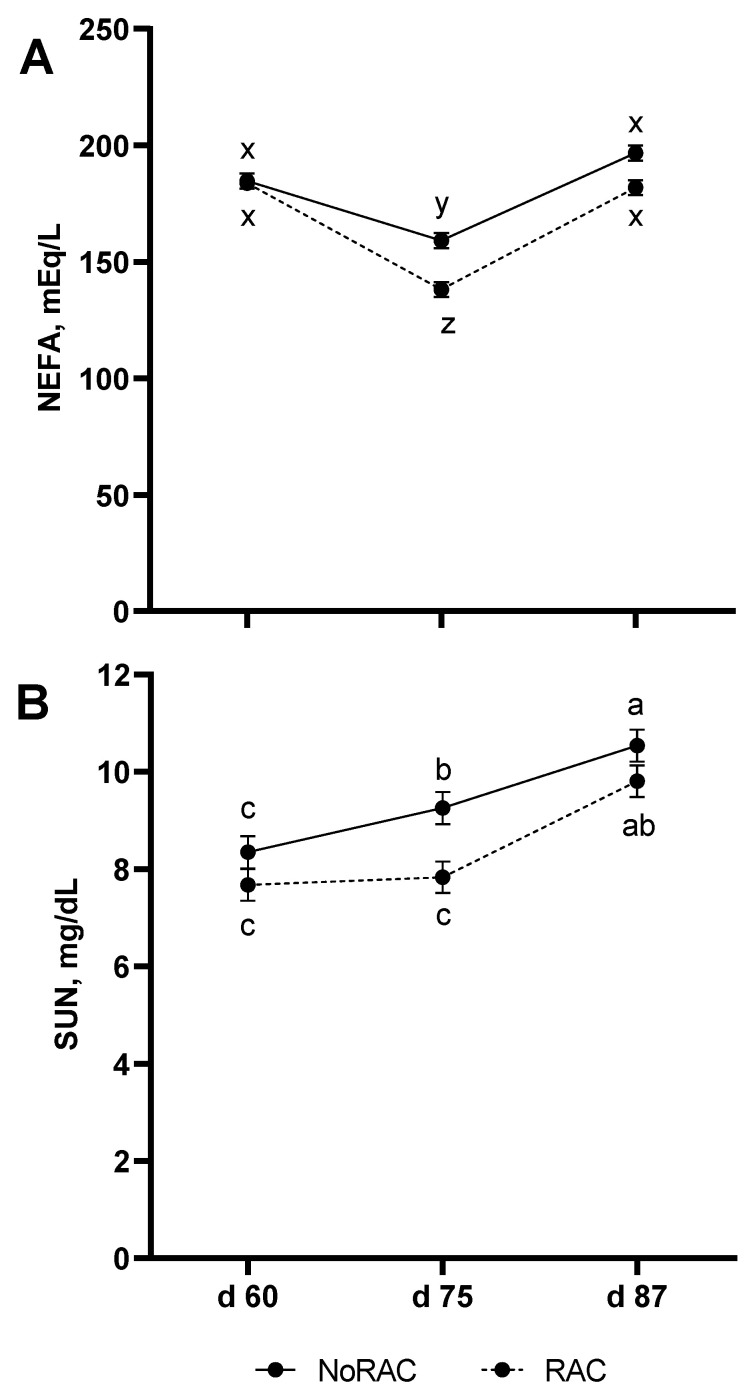
Beta agonist (BA) supplementation and day of sampling influences serum non-esterified fatty acid (NEFA) and serum urea nitrogen (SUN) concentrations. Ractopamine hydrochloride was supplemented for 28 day prior to harvest, starting on day 61, at 0 (NoRAC) or 300 (RAC) mg·steer^−1^·day^−1^. (**A**) Serum NEFA concentrations tended to decrease on day 75 for both BA treatments with lesser concentrations for RAC. Both BA treatments regained initial concentrations by day 87 (BA × Day; *p* = 0.06). Superscripts (x, y, z) that differ tend (*p* < 0.15) to be different. (**B**) Serum urea nitrogen concentrations increased across sampling date for NoRAC, while SUN did not differ for RAC between day 60 and 75 but increased from day 75 to 87 (BA × Day; *p* = 0.05). Superscripts (a, b, and c) are different (*p* < 0.05).

In addition to effects of Cu within BA treatment on glycerol release, the increased availability of cAMP to activate protein kinase A and subsequently hormone sensitive lipase [37,38] would support hydrolysis of triacylglycerides. Thus increasing release of free fatty acids from adipose tissue to be used as energy for the accretion of muscle [39]. Although this would suggest BA activation of this pathway and PDE inhibition through Cu would lead to increased serum NEFA concentrations, a tendency for a BA × Day effect (*p* = 0.06; Figure 2A) was observed in which NEFA concentrations decreased for both NoRAC and RAC steers on day 75 (day 15 of BA period) in comparison to day 60, although RAC serum NEFA was lower on day 75 than NoRAC. By day 87, serum NEFA had returned to initial concentrations for both treatments. The greater depression of NEFA in RAC compared to NoRAC on day 75 is likely due to released NEFA from BA-induced lipolysis being utilized as energy by growing tissues rather than accumulating in the serum. This decrease in serum NEFA due to BA supplementation has previously been observed [40]. No effects of Cu on NEFA (LO: 2.24, MED: 2.23, and HI: 2.24 mEq/L) or SUN (LO:8.79, MED:9.18, and HI:8.76 mg/dL) were observed (*p* ≥ 0.54), in addition to no Cu × BA or Cu × BA × Day effects (*p* ≥ 0.17). However, SUN data displayed a BA × Day effect (*p* = 0.05) in which SUN increased across sampling date (day 60, 75, and 87) in NoRAC steers, while RAC steers did not differ from day 60 to 75 but increased from day 75 to 87 (Figure 2B). Serum urea nitrogen concentrations did not differ between NoRAC and RAC steers on day 60 or 87 but were lesser in RAC on day 75. Interestingly, the steady SUN concentrations between day 60 and 75 in RAC steers suggest less muscle catabolism during the first half of the BA period as SUN increased to a similar concentration as NoRAC by day 87 supporting the disappearance of serum NEFA to fuel muscle growth during this period. However, the diet change at the beginning of the BA period may have also contributed to the difference in blood measurements for NoRAC and RAC steers across this period. Through the shift to more corn by-products in the diet, metabolizable protein (MP) in the diet during the BA period increased by 26%. However, both pre-BA (121% of MP requirement) and BA (147% of MP requirement) diets provided adequate MP based upon observed BW, DMI, and ADG for each respective period calculated by the Beef Ration and Nutrition Decision Software (BRaNDS; Iowa Beef Center at Iowa State University) using NASEM [5] nutrient values for ingredients. This excess protein from the increase in corn by-products may be responsible for the increase in SUN over the course of the BA period for both NoRAC and RAC due to the deamination of protein involved in utilizing protein for energy, resulting in the production of urea as waste [41].

**Figure 3:**
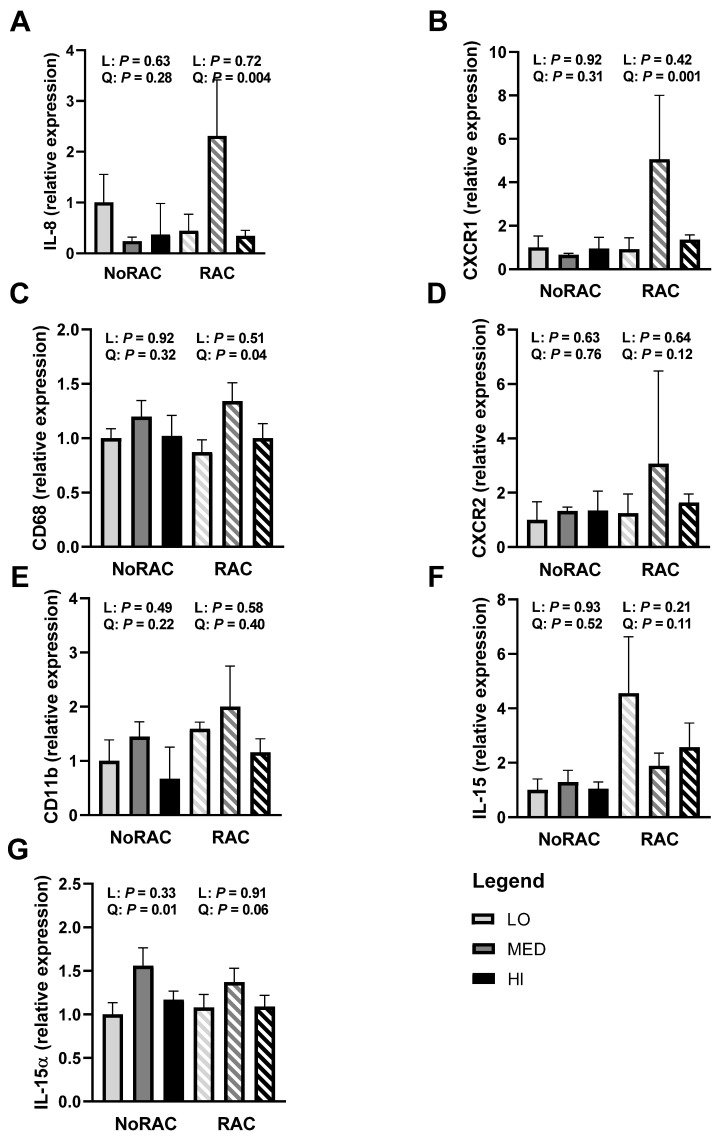
Relative gene expression (quantitative real-time PCR) of markers of inflammation in the muscle from steers fed 0, 10, or 20 mg Cu/kg DM for LO, MED, and HI, respectively and 0 (NoRAC) or 300 (RAC) mg·steer^−1^·day^−1^ of ractopamine hydrochloride. Muscle samples (longissimus thoracis) were collected on day 66 or 67 (day 6 or 7 of RAC feeding). All mRNA relative expression were calculated relative to LO-NoRAC treatment. (**A**) Interleukin-8 (IL-8) gene expression quadratically increased with increasing Cu supplementation within RAC (*p* = 0.004). (**B**) Chemokine C-X-C motif receptor 1 (CXCR1) gene expression quadratically increased with increasing Cu supplementation within RAC (*p* = 0.001). (**C**) Cluster of differentiation 68 (CD68) gene expression quadratically increased within RAC (*p* = 0.04) with MED-RAC having the greatest relative expression. (**D**) Chemokine C-X-C motif receptor 2 (CXCR2) gene expression tended to quadratically increase with increasing Cu supplementation within RAC (*p* = 0.12). (**E**) Cyclin-dependent kinase 11B (CD11B) gene expression was not influenced by linear or quadratic effects of Cu within either NoRAC or RAC (*p* ≥ 0.22). (**F**) Interleukin-15 (IL-15) gene expression tended to quadratically decrease within RAC (*p* = 0.11), with MED-RAC having least gene expression. (**G**) Interleukin-15α (IL-15α) gene expression quadratically increased and tended to quadratically increase with increasing Cu supplementation within NoRAC (*p* = 0.01) and RAC (*p* = 0.06), respectively, with MED having the greatest relative expression. No further linear or quadratic effects of Cu supplementation were observed within NoRAC or RAC for all parameters tested (*p* ≥ 0.21).

Beta agonist supplementation has been linked to inflammation as Genther-Schroeder et al. [22] observed greater serum IL-8 concentrations and shifts in plasma trace mineral concentrations in BA-fed cattle. Although inflammation is often associated with negative connotations, evidence in the basic literature suggests some interleukins, including IL-8 and IL-15, are myokines [42]. Produced locally by the muscle, myokines appear to be important growth factors. A dose-dependent increase in cell proliferation has been observed due to IL-8 treatment in cell culture of human skin and lung cells [43,44,45]. The expression of IL-8 and its receptors CXCR1 and CXCR2 have been correlated with cell proliferation in both prostate [46] and nervous system [47] cells. In the current study, quadratic increases in longissimus thoracis mRNA relative expression of IL-8 (Figure 3A), the IL-8 receptor CXCR1 (Figure 3B), and macrophage marker CD68 (Figure 3C) were observed within RAC (*p* ≤ 0.04). Additionally, IL-8 receptor CXCR2 (Figure 3D) tended to quadratically increase relative gene expression within RAC (*p* = 0.12). These data support the relationship between myokine expression and cattle growth, as greatest expression and performance during the BA feeding period were similarly noted in MED steers. No linear or quadratic effects of Cu within either NoRAC or RAC were observed for the macrophage and neutrophil marker CD11B (Figure 3E; *p* ≥ 0.22). 

Relative mRNA expression of IL-15 (Figure 3F) tended to quadratically decrease within RAC treatment (*p* = 0.11), with MED-RAC having the lowest relative expression. Interestingly, relative expression of the IL-15 receptor IL-15α (Figure 3G) quadratically increased within NoRAC (*p* = 0.01) and tended to quadratically increase within RAC (*p* = 0.06), with MED having greatest relative expression within both BA treatments. No further linear or quadratic effects of Cu were observed within NoRAC or RAC for IL-8, CXCR1, CD68, CXCR2, IL-15, or IL-15α (*p* ≥ 0.21). Considering the affinity of IL-15 to its receptor IL-15α is quite high [48], it is surprising that IL-15 and IL-15α gene expression do not mirror each other in the present study. Interestingly, IL-15 translocation has been observed to be independent of IL-15α under some circumstances [49], thereby suggesting IL-15 and IL-15α gene expression may be independent, in line with our results. Although IL-15 expression did not align with performance trends, its role as a myokine would be beneficial to BA-induced growth. Overexpression of IL-15 in mouse skeletal muscle in cell culture has been observed to increase myoblast differentiation [50], while IL-15 treatment in rats decreased protein degradation [51], both key components of muscle growth. Therefore, additional work to understand the effects of BA on inflammation markers, including IL-15, appears necessary.

Together, the literature gathered here and our preliminary data, although not consistent between IL-8 and IL-15, suggest increased myokine production during BA use may be a key component to the BA anabolic response. Therefore, the quadratic increase in gene expression of IL-8, CXCR1, and CXCR2 within RAC may have supported the quadratic increase in cattle performance observed within RAC. Despite their small sampling size, these data provide preliminary evidence of inflammation occurring due to BA use that may be further influenced by strategic Cu supplementation and the associated high growth rates. More research is warranted to better understand how inflammation, and myokines specifically, may be a vital component to high growth rates in cattle.

## 4. Conclusions

Although limited by sampling size, our data support our objective to examine differential responses in performance, carcass characteristics, and lipolytic rate due to Cu supplementation within cattle receiving NoRAC or RAC. We observed a differential growth response during the BA period due to increasing supplemental Cu in steers receiving RAC in comparison to NoRAC steers. This suggests Cu influences the pathway in which BA elicit a growth response, potentially through the inhibition of PDE indicated by the similarities of the in vitro lipolysis released glycerol and cattle performance during the BA period. Furthermore, these data suggest NASEM [5] recommendations for Cu are adequate for BA induced growth, while greater or lesser concentrations of Cu may cause adverse impacts on cattle performance when utilizing a BA. This effect is likely strongly related to initial Cu status of cattle at the time of BA feeding. Given the critical importance of lipid mobilization and subsequent use as an energy source across all phases of beef and dairy production (including lactation), it is important to further refine Cu status ranges and dietary requirements to optimize cattle performance.

## Figures and Tables

**Table 1:** Ingredient and composition analysis of non-copper-supplemented diet in pre-beta agonist (BA) and BA period.

Ingredient	Pre-BA Period ^1^	BA Period ^2^
	% of Diet DM	% of Diet DM
Dry rolled corn	68.9	62.0
MDGS ^3^	16.7	25.0
Bromegrass hay	8.9	8.0
DDGS ^4^	3.33	3.05
Limestone	1.7	1.5
Salt	0.31	0.31
Vitamin and mineral premix ^5^	0.13	0.13
Rumensin	0.0135	0.0135
Analyzed composition, %		
Crude protein ^6^	12.62	15.31
Neutral detergent fiber ^6^	20.14	19.35
Ether extract ^6^	4.03	4.69
Cu, mg/kg DM ^7^	6.1	6.0
Zn, mg/kg DM ^8^	64.4	72.0
Calculated composition ^9^		
Sulfur, %	0.22	0.27
NEm, Mcal/kg	2.04	2.07
NEg, Mcal/kg	1.38	1.40
ME, Mcal/kg	3.01	3.04

^1^ Pre-BA period, day 0–60. ^2^ BA period, day 61–88. ^3^ Modified distiller’s grains with solubles. ^4^ Dried distiller’s grains with solubles. ^5^ Premix provided 2,200 IU vitamin A and 25 IU vitamin E/kg diet and NASEM (2016) recommendations for Co, Mn, Se, Zn, and I from inorganic sources in addition to 0 (LO), 10 (MED), or 20 (HI) mg Cu/kg DM from Availa Cu (Zinpro Corporation, Eden Prairie, MN, USA). ^6^ Analyses were completed by Dairyland Laboratories (Arcadia, WI). ^7^ Analyzed Cu values represent LO dietary treatment total within the respective period; MED and HI dietary treatments were analyzed at 15.2 and 27.3 mg Cu/kg DM for Pre-BA period and 17.3 and 22.0 mg Cu/kg DM for BA period, respectively. Values were analyzed by inductively coupled plasma optical emission spectrometry (ICP Optima 7000 DV, Perkin Elmer, Waltham, MA, USA). ^8^ Analyzed Zn values represent LO dietary treatment for Pre-BA and BA periods measured by inductively coupled plasma optical emission spectrometry (ICP Optima 7000 DV, Perkin Elmer, Waltham, MA, USA). ^9^ Calculations for sulfur, net energy of maintenance (NEm), net energy of gain (NEg), and metabolizable energy (ME) were made with NASEM (2016) nutrient values of ingredients.

**Table 2:** Forward and reverse primers used for quantitative real-time PCR.

Gene	Accession Number	Strand	Sequence (5′–3′)
CAT-1 ^1^	NM_001034349	Forward	GGTCAACAGCAACTACTACG
		Reverse	TGAACATCCTCTCCATCT
IL-8 ^2^	EU276073.1	Forward	CGCTGGACAGCAGAGCTCACAAG
		Reverse	GCCAAGAGAGCAACAGCCAGCT
CXCR1 ^3^	EF597244.2	Forward	GTCCCCGTGAGATAAGCAC
		Reverse	CAGGTTCAGCAGGTAGACA
CXCR2 ^4^	DQ328664.1	Forward	CAACACTGACCTGCCCTCTA
		Reverse	CAGGTTCAGCAGGTAGACA
IL-15 ^5^	U42433.1	Forward	TTTGAGAAGTACTTCCATCCAG
		Reverse	GAAGTGTTGATGAACATTTGCAC
IL-15α ^6^	XM_005214144.4	Forward	CAGGTCAAGAGTTACAGCATCA
		Reverse	ACTTTCGCGGTCTCGTTAAA
CD11B ^7^	NM_175781.1	Forward	AAACTGGCAGAAAGCAACA
		Reverse	CCAGGAAGACTCTGGAGGA
CD68 ^8^	NM_001045902.1	Forward	CAGCCACAGAACTACCAAGAG
		Reverse	TGGTGGTAGCAGGACTATGA
RPS9 ^9^	NM_001101152.1	Forward	CGCCTCGACCAAGAGCTGAAG
		Reverse	CCTCCAGACCTCACGTTTGTTCC

^1^ Carnitine palmitoyl-CoA transferase-1. ^2^ Interleukin-8. ^3^ Chemokine C-X-C motif receptor 1. ^4^ Chemokine C-X-C motif receptor 2. ^5^ Interleukin-15. ^6^ Interleukin-15α. ^7^ Cyclin-dependent kinase 11B. ^8^ Cluster of differentiation 68. ^9^ Ribosomal protein S9.

**Table 3:** Effect of supplemental copper on copper status and performance of steers during pre-beta agonist period.

Item ^3,4,5^	Copper ^1^			SEM ^6^	Contrast *p*-Values ^2^	
	LO	MED	HI		L	Q
steers (*n*)	24	33	34			
Dietary Cu ^7^, mg/day	75	199	315	4.4	<0.01	0.33
Plasma Cu, mg/L						
Day 0	0.43	0.92	0.98	0.024	0.01	0.01
Day 60	0.90	0.96	0.95	0.032	0.26	0.36
Liver Cu ^8^, mg/kg DM						
Day −23	6	14	53	2.0	0.01	0.06
Day 53	14	166	266	8.7	0.01	0.01
Performance						
Day 0 BW, kg	466	470	473	5.1	0.33	0.91
Day 61 BW, kg	579	586	588	6.9	0.32	0.79
DMI, kg/day	12.1	12.3	12.6	0.23	0.15	0.78
ADG, kg	1.8	1.9	1.9	0.05	0.57	0.72
G:F	0.151	0.152	0.148	0.0034	0.61	0.51

^1^ Trace mineral treatments included LO (no supplemental Cu), MED (2016 recommendation of 10 mg Cu/kg DM), and HI (feedlot consultant recommendations of 20 mg Cu/kg DM; Samuelson et al., 2016). Supplementation of Cu was delivered as Availa Cu (Zinpro Corporation, Eden Prairie, MN, USA), and all other minerals were supplemented at national recommendations utilizing inorganic sources. ^2^ Contrasts analyzed data to determine either a linear (L) or quadratic (Q) relationship among dietary Cu treatments. ^3^ Pre-beta agonist period encompasses day 0 through 60 of trial. ^4^ A 4% pencil shrink was applied to all live body weight (BW) measures used to calculate average daily gain (ADG) and gain-to-feed ratio (G:F). ^5^ Performance data were analyzed with prior trial start weight as covariate. ^6^ Values represent the largest SEM between all treatments. ^7^ Dietary Cu consumption was calculated using total DMI values and not treatment averages. ^8^ Liver Cu analysis: *n* = 12/LO treatment and 18/MED and HI treatment.

**Table 4:** Performance of steers fed varying copper concentrations and ractopamine hydrochloride during beta agonist period.

Beta Agonist ^1^	NoRAC			RAC			SEM ^4^	Contrast *p*-Values ^2^			
Copper ^3^	LO	MED	HI	LO	MED	HI		L	Q	L-R	Q-R
Item ^5^											
steers (*n*)	12	17	16	12	16	18					
BA period ^6^											
Day 61 BW, kg	576	584	580	583	587	595					
Day 88 BW, kg	640	645	642	645	661	662	10.2	0.34	0.62	0.28	0.80
DMI, kg/day	11.7	12.1	12.5	11.8	12.7	12.2	0.38	0.12	0.97	0.45	0.10
ADG, kg	2.39	2.24	2.28	2.33	2.75	2.47	0.100	0.43	0.39	0.27	0.01
G:F	0.206	0.186	0.183	0.198	0.216	0.205	0.0079	0.04	0.34	0.51	0.08
Carcass adjusted ^7,8^											
Day 89 BW, kg	643	643	641	647	661	661	10.7	0.92	0.92	0.31	0.58
Overall DMI, kg/day	11.9	12.1	12.4	12.0	12.5	12.4	0.33	0.19	0.96	0.32	0.38
Overall ADG, kg	2.01	2.04	2.02	2.10	2.14	2.08	0.072	0.87	0.78	0.89	0.56
Overall G:F	0.164	0.164	0.159	0.171	0.168	0.164	0.0005	0.46	0.65	0.28	0.90

^1^ Ractopamine hydrochloride was supplemented 28 days prior to harvest at 0 (NoRAC) or 300 mg·steer^−1^·day^−1^ (RAC). ^2^ Contrasts utilized to analyze this period of data test linear and quadratic relationships in Cu supplementation when RAC was not supplemented (L and Q, respectively) and when RAC was supplemented (L-R and Q-R, respectively). ^3^ Dietary Cu treatments consisted of LO (no supplemental Cu), MED (2016 recommendation of 10 mg Cu/kg DM), and HI (feedlot consultant recommendations of 20 mg Cu/kg DM; Samuelson et al., 2016). Supplementation of Cu was delivered as Availa Cu (Zinpro Corporation, Eden Prairie, MN, USA), and all other minerals were supplemented at national recommendations utilizing inorganic sources. ^4^ Values represent the largest standard error of the mean (SEM) between all treatments. ^5^ A 4% pencil shrink was applied to all live BW measures, including G:F calculations. ^6^ Evaluation of performance data during beta agonist (BA) period, day 61–88. Day 61 BW were included as a reference but were not analyzed as the full factorial due to timing of BA treatment. ^7^ Carcass-adjusted overall performance utilized final BW calculated by dividing hot carcass weight by average dressing percent (62.8%). Overall dry matter intake (DMI) was not influenced by carcass adjustment but was included to provide a concise view of overall parameters in one table. ^8^ Performance data analyzed with prior trial starting weight as covariate.

**Table 5:** The effect of copper supplementation and ractopamine hydrochloride treatment on indicators of copper status and carcass characteristics of beef finishing steers.

Beta Agonist ^1^	NoRAC			RAC			SEM ^4^	Contrast *p*-Values ^2^			
Copper ^3^	LO	MED	HI	LO	MED	HI		L	Q	L-R	Q-R
Item											
steers (*n*)	12	17	16	12	16	18					
Dietary Cu, mg/day	71	194	305	72	201	300	6.8	<0.01	0.39	<0.01	0.03
Plasma Cu, mg/L											
Day 66 or 67	0.94	0.93	0.92	0.90	0.99	0.96	0.042	0.70	0.91	0.31	0.17
Day 87	0.94	1.02	1.06	0.99	1.08	1.08	0.041	0.04	0.66	0.08	0.32
Liver Cu ^5^, mg/kg DM											
Day 89	18	232	289	33	227	324	18.6	0.01	0.01	0.01	0.01
Carcass characteristics ^6^											
Hot carcass weight, kg	404	404	403	406	415	415	6.7	0.92	0.91	0.31	0.58
Dressing percent, %	63.1	62.7	62.8	63.0	62.8	62.7	0.34	0.53	0.51	0.59	0.79
Ribeye area, cm^2^	93.4	91.1	90.9	92.6	98.1	95.5	2.13	0.38	0.65	0.29	0.08
Marbling ^7^	427	463	472	422	458	461	23.4	0.16	0.59	0.21	0.52
Rib fat, cm	1.46	1.46	1.37	1.52	1.45	1.41	0.136	0.61	0.71	0.53	0.87
KPH, %	2.3	2.6	2.5	2.4	2.4	2.5	0.16	0.48	0.23	0.64	0.87
Yield grade	3.15	3.33	3.21	3.30	3.02	3.14	0.194	0.82	0.46	0.52	0.36

^1^ Ractopamine hydrochloride was supplemented 28 days prior to harvest at 0 (NoRAC) or 300 mg·steer^−1^·day^−1^ (RAC). ^2^ Contrasts utilized to analyze this period of data test linear and quadratic relationships in Cu supplementation when RAC was not supplemented (L and Q, respectively) and when RAC was supplemented (L-R and Q-R, respectively). ^3^ Dietary Cu treatments consisted of LO (no supplemental Cu), MED (2016 recommendation of 10 mg Cu/kg DM), and HI (feedlot consultant recommendations of 20 mg Cu/kg DM; Samuelson et al., 2016). Supplementation of Cu was delivered as Availa Cu (Zinpro Corporation, Eden Prairie, MN, USA), and all other minerals were supplemented at national recommendations utilizing inorganic sources. ^4^ Values represent the largest standard error of the mean (SEM) between all treatments. ^5^ Liver Cu analysis: *n* = 6/LO treatment and 9/MED and HI treatment. ^6^ Initial weights from prior trial were used as covariate for carcass data analysis. ^7^ Marbling scores: slight = 300, small = 400, modest = 500, moderate = 600, slightly abundant = 700, and moderately abundant = 800.

## Data Availability

The data presented in this study are available on request from the corresponding author. The data are not publicly available due to funding agency restrictions.

## References

[1] Suttle N.F. (2010). The Mineral Nutrition of Livestock.

[2] Suttle N.F. (1991). The interactions between copper, molybdenum, and sulphur in ruminant nutrition. Annu. Rev. Nutr..

[3] Arthington J. (2003). Copper antagonists in cattle nutrition. Proceedings of the 14th Annual Florida Ruminant Nutrition Symposium.

[4] Bidewell C.A., Drew J.R., Payne J.H., Sayers A.R., Higgins R.J., Livesey C.T. (2012). Case study of copper poisoning in a British dairy herd. Vet. Rec..

[5] NASEM (2016). Nutrient Requirements of Beef Cattle.

[6] Samuelson K.L., Hubbert M.E., Galyean M.L., Löest C.A. (2016). Nutritional recommendations of feedlot consulting nutritionists: The 2015 New Mexico State and Texas Tech University survey. J. Anim. Sci..

[7] Kincaid R.L. (2000). Assessment of trace mineral status of ruminants: A review. J. Anim. Sci..

[8] Rucker R.B., Kosonen T., Clegg M.S., Mitchell A.E., Rucker B.R., Uriu-Hare J.Y., Keen C.L. (1998). Copper, lysyl oxidase, and extracellular matrix protein cross-linking. Am. J. Clin. Nutr..

[9] Keller G.A., Warner T.G., Steimert K.S., Hallewell R.A. (1991). Cu,Zn superoxide dismutase is a peroxisomal enzyme in human fibroblasts and hepatoma cells. Proc. Natl. Acad. Sci. USA.

[10] Johnson B.J., Smith S.B., Chung K.Y. (2014). Historical overview of the effect of β-adrenergic agonists on beef cattle production. Asian-Australas. J. Anim. Sci..

[11] Krishnamoorthy L., Cotruvo J.A., Chan J., Kaluarachchi H., Muchenditsi A., Pendyala V.S., Jia S., Aron A.T., Ackerman C.M., Vander Wal M.N. (2016). Copper regulates cyclic-AMP-dependent lipolysis. Nat. Chem. Biol..

[12] VanValin K.R., Genther-Schroeder O.N., Laudert S.B., Hansen S.L. (2019). Relative bioavailability of organic and hydroxy copper sources in growing steers fed a high antagonist diet. J. Anim. Sci..

[13] Engle T.E., Spears J.W. (2000). Effects of dietary copper concentration and source on performance and copper status of growing and finishing steers. J. Anim. Sci..

[14] Koltes D.A., Spurlock D.M. (2011). Coordination of lipid droplet-associated proteins during the transition period of Holstein dairy cows. J. Dairy Sci..

[15] Pampusch M.S., Johnson B.J., White M.E., Hathaway M.R., Dunn J.D., Waylan A.T., Dayton W.R. (2003). Time course of changes in growth factor mRNA levels in muscle of steroid-implanted and nonimplanted steers. J. Anim. Sci..

[16] Pothoven M.A., Beitz D.C., Thornton J.H. (1975). Lipogenesis and lipolysis in adipose tissue of ad libitum and restricted-fed beef cattle during growth. J. Anim. Sci..

[17] National Institute of Standards and Technology (NIST) (2014). NIST/EPA/NIH Mass Spectral Library (NIST 14) and NIST Mass Spectral Search Program (Version 2.2).

[18] McGill J.L., Rusk R.A., Guerra-Maupome M., Briggs R.E., Sacco R.E. (2016). Bovine gamma delta T cells contribute to exacerbated IL-17 production in response to co-infection with bovine RSV and Mannheimia haemolytica. PLoS ONE.

[19] Livak K.J., Schmittgen T.D. (2001). Analysis of relative gene expression data using real-time quantitative PCR and the 2^-ΔΔCT^ method. Methods.

[20] Richter E.L., Drewnoski M.E., Hansen S.L. (2012). Effects of increased dietary sulfur on beef steer mineral status, performance, and meat fatty acid composition. J. Anim. Sci..

[21] Pogge D.J., Hansen S.L. (2013). Supplemental vitamin C improves marbling in feedlot cattle consuming high sulfur diets. J. Anim. Sci..

[22] Genther-Schroeder O.N., Branine M.E., Hansen S.L. (2016). The effects of increasing supplementation of zinc-amino acid complex on growth performance, carcass characteristics, and inflammatory response of beef cattle fed ractopamine hydrochloride. J. Anim. Sci..

[23] Genther-Schroeder O.N., Branine M.E., Hansen S.L. (2016). The influence of supplemental Zn-amino acid complex and ractopamine hydrochloride feeding duration on growth performance and carcass characteristics of finishing beef cattle. J. Anim. Sci..

[24] Feldpausch J.A., Amachawadi R.G., Scott H.M., Tokach M.D., Dritz S.S., Woodworth J.C., Nagaraja T.G., Goodband R.D., DeRouchey J.M. (2015). Effects of added copper and zinc on growth performance and carcass characteristics of finishing pigs fed diets with or without ractopamine HCl. Kans. Agric. Exp. Stn. Res. Rep..

[25] Ward J.D., Spears J.W., Kegley E.B. (1993). Effect of copper level and source (copper lysine vs copper sulfate) on copper status, performance, and immune response in growing steers fed diets with or without supplemental molybdenum and sulfur. J. Anim. Sci..

[26] Engle T.E., Spears J.W., Xi L., Edens F.W. (2000). Dietary copper effects on lipid metabolism and circulating catecholamine concentrations in finishing steers. J. Anim. Sci..

[27] Engle T.E., Spears J.W. (2001). Performance, carcass characteristics, and lipid metabolism in growing and finishing Simmental steers fed varying concentrations of copper. J. Anim. Sci..

[28] Gooneratne S.R., Buckley W.T., Christensen D.A. (1989). Review of copper deficiency and metabolism in ruminants. Can. J. Anim. Sci..

[29] Zinn R.A., Alvarez E., Mendez M., Montañ M., Ramirez E., Shen Y. (1997). Influence of dietary sulfur level on growth performance and digestive function in feedlot cattle. J. Anim. Sci..

[30] Engle T.E., Spears J.W. (2000). Dietary copper effects on lipid metabolism, performance, and ruminal fermentation in finishing steers. J. Anim. Sci..

[31] Odiet J.A., Boerrigter M.E., Wei J.Y. (1995). Carnitine palmitoyl transferase-I activity in the aging mouse heart. Mech. Ageing Dev..

[32] Song M., Schuschke D.A., Zhou Z., Chen T., Pierce W.M., Wang R., Johnson W.T., McClain C.J. (2012). High fructose feeding induces copper deficiency in Sprague–Dawley rats: A novel mechanism for obesity related fatty liver. J. Hepatol..

[33] Lei L., Xiaoyi S., Fuchang L. (2017). Effect of dietary copper addition on lipid metabolism in rabbits. Food Nutr. Res..

[34] Engle T.E., Spears J.W., Armstrong T.A., Wright C.L., Odle J. (2000). Effects of dietary copper source and concentration on carcass characteristics and lipid and cholesterol metabolism in growing and finishing steers. J. Anim. Sci..

[35] Johnson L.R., Engle T.E. (2003). The effects of copper source and concentration on lipid metabolism in growing and finishing Angus steers. Asian-Australas. J. Anim. Sci..

[36] Balkin M.S., Sonenberg M. (1981). Hormone-induced homologous and heterologous desensitization in the rat adipocyte. Endocrinology.

[37] Beavo J.A., Bechtel P.J., Krebs E.G. (1974). Activation of protein kinase by physiological concentrations of cyclic AMP. Proc. Natl. Acad. Sci. USA.

[38] Mersmann H.J. (1998). Overview of the effects of beta-adrenergic receptor agonists on animal growth including mechanisms of action. J. Anim. Sci..

[39] Yeaman S.J. (1990). Hormone-sensitive lipase-a multipurpose enzyme in lipid metabolism. Biochim. Biophys. Acta (C) Mol. Cell Res..

[40] Carmichael R.N. (2019). The Influence of Dietary Zinc Concentration during Periods of Rapid Growth Induced by Ractopamine Hydrochloride or Dietary Energy Content on Trace Mineral Metabolism and Performance of Beef Steers. Master’s Thesis.

[41] Klopfenstein T.J., Erickson G.E., Bremer V.R. (2008). Board-invited review: Use of distillers by-products in the beef cattle feeding industry. J. Anim. Sci..

[42] Pedersen B.K., Åkerström T.C., Nielsen A.R., Fischer C.P. (2007). Role of myokines in exercise and metabolism. J. Appl. Physiol..

[43] Tuschil A., Lam C., Haslberger A., Lindley I. (1992). Interleukin-8 stimulates calcium transients and promotes epidermal cell proliferation. J. Investig. Dermatol..

[44] Rennekampff H.O., Hansbrough J.F., Kiessig V., Doré C., Sticherling M., Schröder J.M. (2000). Bioactive interleukin-8 is expressed in wounds and enhances wound healing. J. Surg. Res..

[45] Luppi F., Longo A.M., de Boer W.I., Rabe K.F., Hiemstra P.S. (2007). Interleukin-8 stimulates cell proliferation in non-small cell lung cancer through epidermal growth factor receptor transactivation. Lung Cancer.

[46] Murphy C., McGurk M., Pettigrew J., Santinelli A., Mazzucchelli R., Johnston P.G., Montironi R., Waugh D.J. (2005). Nonapical and cytoplasmic expression of interleukin-8, CXCR1, and CXCR2 correlates with cell proliferation and microvessel density in prostate cancer. Clin. Cancer Res..

[47] Sharma I., Singh A., Siraj F., Saxena S. (2018). IL-8/CXCR1/2 signalling promotes tumor cell proliferation, invasion and vascular mimicry in glioblastoma. J. Biomed. Sci..

[48] Lorenzen I., Dingley A.J., Jacques Y., Grötzinger J. (2006). The structure of the interleukin-15α receptor and its implications for ligand binding. J. Biol. Chem..

[49] Duitman E.H., Orinska Z., Bulanova E., Paus R., Bulfone-Paus S. (2008). How a cytokine is chaperoned through the secretory pathway by complexing with its own receptor: Lessons from interleukin-15 (IL-15)/IL-15 receptor α. Mol. Cell. Biol..

[50] Quinn L.S., Anderson B.G., Drivdahl R.H., Alvarez B., Argilés J.M. (2002). Overexpression of interleukin-15 induces skeletal muscle hypertrophy in vitro: Implications for treatment of muscle wasting disorders. Exp. Cell Res..

[51] Carbó N., López-Soriano J., Costelli P., Busquets S., Alvarez B., Baccino F.M., Quinn L.S., López-Soriano F.J., Argilés J.M. (2000). IL 15 antagonizes muscle protein waste in tumour-bearing rats. Br. J. Cancer.

